# Association of NEF2L2 Rs35652124 Polymorphism with Nrf2 Induction and Genotoxic Stress Biomarkers in Autism

**DOI:** 10.3390/genes14030718

**Published:** 2023-03-15

**Authors:** Lev N. Porokhovnik, Vladimir M. Pisarev, Anastasia G. Chumachenko, Julia M. Chudakova, Elizaveta S. Ershova, Natalia N. Veiko, Natalia L. Gorbachevskaya, Uliana A. Mamokhina, Alexander B. Sorokin, Anna Ya. Basova, Mikhail S. Lapshin, Vera L. Izhevskaya, Svetlana V. Kostyuk

**Affiliations:** 1 Research Centre for Medical Genetics, 1 Moskvorechie Street, 115478 Moscow, Russia; 2 Federal Research and Clinical Center of Intensive Care Medicine and Rehabilitology, 25 Petrovka Street, 107031 Moscow, Russia; 3 Mental Health Research Center, 34 Kashirskoe highway, 115478 Moscow, Russia; 4 Federal Resource Center for Organization of Comprehensive Support to Children with Autism Spectrum Disorders, 29 Sretenka Street, 127051 Moscow, Russia; 5 Haskins Laboratories, 300 George Street, New Haven, CT 06511, USA; 6 G.E. Sukhareva Research and Practical Center of Children and Adolescents Mental Health, 21A Fifth Donskoy Drive, 119334 Moscow, Russia

**Keywords:** Nrf2, NFE2L2, autism, ASD, polymorphism, SNP, dimethyl fumarate, antioxidant, oxidative stress, H2AX, rs35652124

## Abstract

Increased oxidative/genotoxic stress is known to impact the pathophysiology of ASD (autism spectrum disorder). Clinical studies, however, reported limited, heterogeneous but promising responses to treatment with antioxidant remedies. We determined whether the functional polymorphism of the Nrf2 gene, master regulator of anti-oxidant adaptive reactions to genotoxic stress, links to the genotoxic stress responses and to an in vitro effect of a NRF2 inductor in ASD children. Oxidative stress biomarkers, adaptive responses to genotoxic/oxidative stress, levels of master antioxidant regulator Nrf2 and its active form pNrf2 before and after inducing by dimethyl fumarate (DMF), and promotor rs35652124 polymorphism of NFE2L2 gene encoding Nrf2 were studied in children with ASD (*n* = 179). Controls included healthy adults (*n* = 101). Adaptive responses to genotoxicity as indicated by H2AX and cytoprotection by NRF2 contents positively correlated in ASD children with a Spearman coefficient of R = 0.479 in T+, but not CC genotypes. ASD children with NRF2 rs35652124 CC genotype demonstrated significantly higher H2AX content (0.652 vs. 0.499 in T+) and pNrf2 induction by DMF, lowered 8-oxo-dG concentration in plasma and higher cfDNA/plasma nuclease activity ratio. Our pilot findings suggest that in ASD children the NEF2L2 rs35652124 polymorphism impacts adaptive responses that may potentially link to ASD severity. Our data warrant further studies to reveal the potential for NEF2L2 genotype-specific and age-dependent repurposing of DMF and/or other NRF2-inducing drugs.

## 1. Introduction

The term “autism spectrum disorder” (ASD) encompasses heterogeneous neurodevelopmental conditions characterized by delayed development, early-onset deficits in social communication, and restricted, repetitive sensory-motor behavior of variable significance [1]. The causes of ASD include a complex combination of genetic and environmental factors and birth-related parameters that may act in concert during the development of the neural system [2,3]. Although the pathophysiology of psychiatric diseases, including ASD, is far from being fully elucidated, a growing body of clinical and preclinical evidence has discovered the pivotal role of oxidative stress in their pathogenesis [4,5,6]. The studies in this field have indicated that various psychiatric diseases are characterized by oxidation disbalance exhibiting higher levels of oxidative biomarkers and lower levels of antioxidant defense biomarkers in the brain and peripheral tissues. The resultant DNA damage and deficient repair of oxidative DNA lesions have been proposed to contribute to the development of schizophrenia and ASD [7]. The elevated free radicals result in genotoxicity and launch a cascade of sterile inflammation and microglial activation in the affected brain [8]. Oxidative stress, increased inflammation as a hallmark of a dysregulated immune response, and abnormal mitochondrial metabolism are inextricably linked, seemingly representing the common molecular underpinning of many neuropathies [9], including ASD. In most cases, antioxidant therapy yields small or no effect. Studying molecular pathways underpinning the major pathogenic clues to the cellular-level regulation of genotoxic stress and adaptive antioxidant shielding in norm and ASD has recently become a promising avenue to reveal novel approaches to the personalization of ASD management and correction.

In response to oxidative/genotoxic stress, the cells initiate the expression of a battery of protective genes encoding antioxidant enzymes and transporters. A coordinated launch of transcription of the cell defense genes is managed by some transcription factors. Nrf2 (nuclear E2-related factor 2) protein is a major transcription factor sensible to the shifts of reductive-oxidative status [10]. After the induction by increased FR level, Nrf2 penetrates the nucleus and launches the expression of 100–200+ genes that contain a special motif in the promotor region, so-called ARE (antioxidant response element) to bind Nrf2: 5′-A/GTGAC//nnnGCA/G-3′ [11]. The ARE-driven genes include genes for antioxidant enzymes (heme oxygenase 1, superoxide dismutase 1, glutathione peroxidase 2, glutamate-cysteine ligase, glutathione reductase, thioredoxin reductase, etc.) and phase II xenobiotic detoxification enzymes (glutathione-S-transferase A, M, P, NAD(P)H:quinone oxidoreductase 1, NRH:quinone oxidoreductase 2, UDP-glucuronosyltransferase A and B, etc.) [12]. Thus, Nrf2 should reduce genotoxicity caused by xenobiotics and free radicals.

NFE2L2 gene encoding Nrf2 transcription factor is located on human chromosome 2. In 2013, the genetic databases contained 583 allelic forms of NFE2L2, of which 18 single nucleotide polymorphism (SNP) variants were associated with the risk of a particular disease. The involved diseases include respiratory diseases and critical conditions [13,14], male infertility [15], cardiovascular and pulmonary diseases [16], diabetes [17], gastrointestinal diseases [18], and neurodegenerative diseases such as Parkinson’s disease [19,20], Alzheimer’s disease [21], amyotrophic lateral sclerosis [22]. All these disorders share oxidative stress and elevated cell death, which are also intrinsic to ASD.

To decrease central oxidative and nitrosative stress, alleviate neuroinflammation, unfolded protein response and endoplasmic reticular stress that all result in genotoxicity and impairment of neural cell functionality and apoptosis, various Nrf2 activators have been proposed in human [23] and animal model settings (see also ClinicalTrials.gov ID NCT01894958). No studies have been performed to personalize potential ASD treatment strategies to enhance NRF2 signaling based on genetic polymorphism. Our study attempted for the first time to link these treatments to NRF2 functional genetic polymorphism in an in vitro model of ASD treatment using the pharmacological NRF2 inductor dimethyl fumarate (DMF). The latter represents a subscription drug to treat Parkinson’s disease and a candidate drug under evaluation for Alzheimer’s disease, Huntington’s disease, and amyotrophic lateral sclerosis [24,25,26]. We believe that thorough justification of re-purposing of the DMF drug to treat ASD on a personalized base is currently highly desirable.

The aim of the study was to examine the eventual contribution of functional NRF2 polymorphism to pathogenically significant candidate markers of adaptive responses to genotoxic stress potentially intrinsic to ASD. To that end, we chose a promotor rs35652124 polymorphic site of the NFE2L2 gene that encodes Nrf2 protein based on the proven role of this SNP in the progression of neuropsychiatric disorders and other diseases (see “Discussion”), and determined the frequencies of each allele in a group of children with ASD and healthy volunteers from the same population (Moscow, Russia).

In the group of ASD children, we determined the values of Nrf2 expression in lymphocytes at the levels of mRNA and protein product for the purpose of discovering a possible association between the allelic polymorphism and the expression levels of NRF2 and its phosphorylated form (phospho-NRF2) and integral indices of genotoxicity, oxidative stress and cell death (8-oxo-dG DNA modification and expression of H2AX in the immune system cells).

Finally, we tried to determine if there is a dependence of the level of NRF2 induction by the specific inducer DMF on the Nrf2 rs35652124 polymorphism in the lymphocytes of children with ASD in vitro.

## 2. Materials and Methods

### 2.1. Study Cohorts

The Declaration of Helsinki and the International Conference on Harmonization (ICH) Good Clinical Practice (GCP) Guideline (1998) have been employed to develop a Protocol for this particular study that has received approval from the Ethics Committee of the G.E. Sukhareva Research and Practical Center of Children and Adolescents Mental Health, Moscow Department of Public Health. The Ethics Committee decision number is #3-Jun-27-2017 (dd. 27 June 2017).

The ASD cases were recruited from day patients who had been admitted to the preschool division of G.E.Sukhareva Research and Practical Center of Children and Adolescents Mental Health (Moscow, Russia) and Federal Resource Center for Organization of Comprehensive Support to Children with Autism Spectrum Disorders (Moscow, Russia). The clinical group included 181 Caucasian children and adolescents aged 3 to 18 with proven ASD.

ASD was diagnosed using the criteria established by the DSM-5. Additionally, the patients underwent the following tests:

1. Autism Mental Status Examination (AMSE) is an 8-item observational assessment that prompts the examiner to observe and document patients’ social, communicative, and behavioral functioning in the context of a routine clinical examination. The AMSE was developed by psychiatrists with autism expertise and is intended to guide clinical judgment in the context of diagnostic decision-making.
2. Childhood Autism Rating Scale (CARS) is a scale for the quantification of the severity of autism pathology. The CARS assesses the child on a scale from 1 to 4 in each of 15 dimensions or symptoms (relating to people; emotional response; imitation; body use; object use; listening response; fear or nervousness; verbal communication; non-verbal communication; activity level; level and reliability of intellectual response; adaptation to change; visual response; taste, smell and touch response; and general impressions). Total scores of or above 30 strongly suggest the presence of autism. Children who have a score from 30 to 36 have mild to moderate autism, while those with scores ranging from 37 to 60 points have severe autism.
3. Communication Questionnaire (SCQ) is a parent questionnaire designed for detecting risk for ASD. The SCQ was originally designed as a screening tool for children 4 years of age or older enrolled in epidemiological research or for studies comparing individuals with ASD and other clinical groups.

Exclusion criteria were (1) neurodevelopmental disorders of known etiology (Rett syndrome, fragile X syndrome, or tuberous sclerosis, etc.); (2) clinically significant sensory or motor impairment; (3) significant medical conditions known to affect brain development (neonatal brain damage, genetic and/or metabolic syndromes involving the CNS, severe nutritional or psychological deprivation); (4) and a history of inflammatory disorders and allergies.

The control group to determine the NFE2L2 allelic frequencies in the general population was formed by 101 adult healthy volunteers who were not relatives of the cases.

### 2.2. Blood Sampling

The biological material sampled for testing was venous blood in the amount of 3–4 mL collected from each participant. DNA for genetic typing was isolated from whole blood using Diatom DNA Prep 200 kits pursuant to the instruction enclosed (Isogene, Moscow, Russia).

### 2.3. SNP Selection Criteria

When choosing the polymorphism site to be studied, we were guided by three principles. First, minor allele frequency (MAF) should be high enough (≥15%) to ensure representativeness and statistical reliability with a realistic sample size (before the study started, we had found no available data with regard to the Russian population, therefore we had considered the control data of European, American, and Australian Caucasian subjects available in free access in [27]). Second, several published reports should demonstrate the clinical significance of the allele in the pathogenesis of some diseases (see “Discussion”). Six polymorphic variants met this criterion: rs6726395, rs7557529, rs35652124, rs2886162, rs10183914, rs1806649. Finally, the selected SNP should directly affect the Nrf2 expression level, hence we primarily focused on the promotor area searching for mutations with proven affected gene expression status.

Based on the above-mentioned criteria, we have chosen one NFE2L2 SNP site: rs35652124, because, unlike the other five variants: (a) the mutation is localized within a promotor region; (b) it associates with the age of Parkinson’s disease onset [19,20,28], as well as with various other diseases in which an oxidation-anti-oxidation balance may pathogenically contribute to the course of the disease [29,30]; and (c) there is multiple evidence that this polymorphism is functional and the alternative alleles associate with distinct levels of NRF2 mRNA [15,31,32] (Table 1).

Owing to the selection of the frequent minor allele, it became possible to use data obtained from a sample of adults, not children, as the local controls for comparing with the childhood patients. As judged by the infant mortality in Russia declared for the current year at a relatively low level of 4.997 deaths per 1000 live births [35], it is apparent that even in case of strong negative selection against a particular allele, lethality in infantile age and childhood cannot change the allele frequency in adult population compared to infants.

### 2.4. Polymerase Chain Reaction

The following primers were synthesized for NRF2 rs35652124:

1. Direct external (F1)—5′-GTCGCTGGAGTTCGGACGCTT-3′,
2. Reverse external (R1)—5′-GCTTTGGTGGGAAGAGGTTCT-3′,
3. Direct internal (F2)—5′-TCGCAGTCACCCTGAACGCCCT-3′,
4. Reverse internal (R2)—5′-AGACACGTGGGAGTTCAGAGGG-3′.

**Figure 1:**
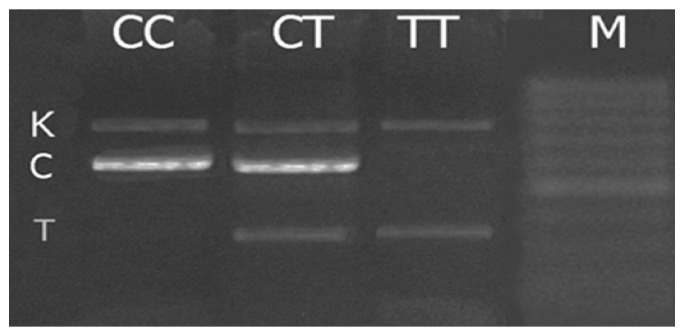
Electrophoregram of the NRF2 rs35652124 amplification products. K—amplification control, 464 bp, C—allele C, 336 bp, T—allele T, 171 bp.

DNA genotyping was performed using tetra-primer polymerase chain reaction (PCR) with GenPak PCR MasterMix Core (Isogene, Moscow, Russia). PCR was carried out in a programmed thermostat GenAmp 9700 (Applied Biosystems, Waltham, MA, USA). The amplification products were separated using electrophoresis in 2% agarose gel followed by visualizing under UV-light (Figure 1).

### 2.5. Flow Cytometry

Flow cytometry was applied to measure the 8-oxo-deoxyguanosine fraction in nuclear DNA with primary monoclonal antibodies (SC-66036, Santa Gruz, CA, USA) and secondary antibodies (anti-mouse-FITC, SC-2010, Santa Gruz, CA, USA), double-stranded DNA break degree using antibodies to histones H2AX (NB100-78356G, NovusBio, Englewood, CO, USA) conjugated with DyLight488, as well as expression levels of proteins Nrf2 and its phosphorylated form using anti-NRF-2 antibodies conjugated with Alexa Fluor^®^ 488. Ex: 495 nm, Em: 519 nm (ab194984), and rabbit antibodies to the phosphorylated form NRF-2 (ser40) (bs2013), and secondary anti-rabbit antibodies conjugated with FITC (Sc 2012).

### 2.6. Evaluation of Gene Expression Using Real-Time PCR

Gene expression was evaluated with RT-PCR [36]. PCR was performed with the corresponding primers (Synthol, Moscow, Russia) and intercalating SybrGreen dye at StepOnePlus device (Applied Biosystems, San Francisco, CA, USA). As a reference gene expressing in human leucocyte, the housekeeping gene TBP, which codes for a protein binding to TATA motifs (TATA-box Binding Protein), was chosen as the most stably expressed gene in human leukocyte culture [37,38].

RNA was isolated from test specimens (peripheral blood lymphocytes of children with ASD) using YellowSolve kits (Clonogen, Saint Petersburg, Russia), or Trizol reagent (Invitrogen) according to the standard technique [39] followed by phenol-chloroform extraction and precipitation with chloroform and isoamyl alcohol (49:1), or applied RNeasy Mini Kits (“Qiagen”, Hilden, Germany) followed by DNAse I treatment and reverse transcription using Reverse Transcriptase Kit (“Silex”, Russia).

We used the following primers (Synthol, Moscow, Russia) (written in the same order (F;R)):

TBP (reference gene) (F: 5′-GCCCGAAACGCCGAATAT-3′; 

R: 5′-CCGTGGTTCGTGGCTCTCT-3′);

NRF2 (TCCAGTCAGAAACCAGTGGAT; GAATGTCTGCGCCAAAAGCTG);

NQO1 (AGCGAGTGTTCATAGGAGAGT, GCAGAGAGTACATGGAGCCAC)

RNA content was determined with dye Quant-iT RiboGreen RNA reagent (MoBiTec, Goettingen, Germany) using the flatbed spectrofluorimeter (PerkinElmer Finland Oy, Turku, Finland) at λ_excit_ = 487 nm, λ_fluor_ = 524 nm. Reverse transcription reaction was carried out using chemicals (Sileks, Moscow, Russia) according to the manufacturer’s protocols. 

The composition of PCR mix per 25 μL: 2.5 μL PCR-buffer (700 mM/L Tris-HCl, pH 8.6; 166 mM/L ammonia sulphate, 35 mM/L MgCl_2_), 2 μL 1.5 mM/L dNTP solution; 1 μL of 30 pcM/L primer solution for each primer, cDNA. PCR conditions were individually selected for each primer pair. The standard for most primers were the following conditions: after denaturation (95 °C, 4 min), 40 amplification cycles were conducted in a regime as follows: 94 °C–20 s, (56–62) °C–30 s, 72 °C–30 s. Then, 72 °C for 5 min. PCR was performed using the StepOnePlus device (Applied Biosystems, Foster City, CA, USA).

Gene expression levels were analyzed in several independent experiments. The data processing was performed using the in-built software with a relative error of 2%.

### 2.7. Extraction of cfDNA Fragments from Plasma and cfDNA Quantification

Cells were extracted from the blood containing heparin by means of centrifuging at 460× *g*, followed by mixing of 1 mL of the blood plasma with 0.1 mL of a solution containing 10% of sodium lauryl sarcosylate, 0.2 M EDTA, and 0.075 mg/mL of RNAse A (Sigma-Aldrich, Saint Louis, MO, USA); incubation for 45 min; and treatment with proteinase K (0.2 mg/mL, Promega, Madison, WI, USA) for 24 h at 37 °C. After two purification cycles using the saturated phenolic solution, the DNA fragments were precipitated by adding two volumes of ethanol in the presence of 2 M ammonium acetate. The precipitate was then washed twice with 75% ethanol, dried, and dissolved in water. The DNA concentration was determined by means of measuring the fluorescence intensity after DNA staining with RiboGreen dye (Molecular Probes/Invitrogen, Carlsbad, CA, USA).

### 2.8. Measurement of DNase1 Activity

The DNAse1 activity was measured using a protocol as described previously [40]. The substrate for the DNAse1 was synthesized by ‘Syntol’, Russia. The substrate was a double-strand oligodeoxyribonucleotide with a 30-base-pair-sequence, i.e., R6G—ACC CCC AGC GAT TAT CCA AGC GGG-BHQ1. At the 5′-end, the oligonucleotide comprised a fluorescent group. As a result of the endonuclease hydrolysis, elevated dye’s fluorescence was registered. Actually, 10 μL of the blood plasma from a particular sample were added to 90 μL of the DNAse1 solution (10 mM HEPES, pH 7.5, 20 mM MgCl_2_, 5 mM CaCl_2_), which contained 3 pM of the oligonucleotide as a substrate. The reaction lasted for 1 h at 37 °C, and the changes in the dye’s fluorescence signal were detected using an Enspire microplate reader (PerkinElmer Finland Oy, Turku, Finland). In oMadison, WI rder to calculate the DNAse1 activity, a calibration curve was plotted, which linked the dye’s fluorescence build-up value with the concentration of a standard DNAse 1 sample (Sigma-Aldrich, MO, USA) in the solution. The activity measurement results are provided in the enzyme units (EU). One EU (ng/mL) corresponds to the activity of the standard DNAse1 solution having a concentration of 1 ng/mL (measured for 1 h at 37 °C). For each sample, not less than three simultaneous measurements were performed with a resultant relative standard error of 5%.

### 2.9. Statistical Analysis

Statistical data processing and analysis were conducted using the software package PAST v. 2.17c [41].

Besides, the results were statistically processed with the help of the InStat GraphPad software package (GraphPad Software Inc., La Jolla, CA, USA): for binary values, Fisher’s exact tests or chi-square tests were applied followed by calculating *p*-value with a 95% confidence interval, while quantitative parameters were compared using Mann-Whitney tests and Student’s *t*-tests. For correlation tests, Spearman rank correlation was calculated. The standard *p*-value to reject a null hypothesis was *p* < 0.05.

Before starting statistical analysis, each sample was tested for anomalous errors (outliers) using Grubbs’s test with the help of an online ‘Outlier calculator’ by GraphPad Software [42]. One anomalous observation of NRF2 protein level was excluded.

The allele frequency distribution was tested for compliance with Hardy-Weinberg equilibrium using the χ^2^ test without Yates adjustment.

## 3. Results

### 3.1. Nrf2 rs35652124 Genotype Frequencies in Children with ASD and Healthy Controls

The frequencies of rs35652124 polymorphic variants of the Nrf2 gene in children with ASD and healthy adult volunteers (control) are shown in Table 2. The comparisons of frequencies of NFE2L2 allelic variants in ASD children (*n* = 179) and the control group (*n* = 101) showed no statistically significant difference.

### 3.2. Adaptive Responses for Genotoxic Stress and Their Modulation with NRF2 Inducer in ASD Children Mononuclear Blood Cells

Next, in a limited cohort of age- and sex-matched ASD children (*n* = 24), selected randomly from the total sample of ASD subjects, we determined the levels of genotoxicity marker, cell-free DNA (cfDNA), and nuclease activity in plasma, the concentration of adaptive response proteins, H2AX, NRF2 and pNRF2, in nuclei of PBMC, and evaluated the potential for modulation of adaptive responses pharmacological NRF2 inducer DMF in vitro in cells from NRF2 rs35652124 genotyped ASD children,

Table 3 shows no difference in the content of NRF2 and pNRF2 proteins in cells from children of alternative NRF2 genotypes. However, the content of H2AX protein, blood nuclease activity, and cell-free DNA concentration significantly differed between carriers of CC and T+ (CT + TT) Nrf2 rs35652124 genotypes: ASD children with genotype CC rs35652124 exhibited significantly lower 8-oxo-G level, and increased cfDNA/blood plasma nuclease activity ratio (reduced during chronic oxidative stress [43,44]) (Table 3).

As shown in Table 3, when analyzing different genotypes apart, cells with allele C in homozygous state in rs35652124 (CC) demonstrated a higher increment of the content of pNrf2 after the exposure to DMF against the background than carriers of genotypes T+ (CT and TT), with almost doubling after induction (*p* = 0.049, see Table 4). It is worthy of notice that Grubbs’s test for outliers was performed on pNRF2 expression data at the protein level. The test resulted in the deletion of one outlying case. After deletion, the distribution has become normal (Gaussian), corroborating the correctness of outlier deletion. Power of the test was 0.883, which exceeds the recommended threshold value (0.8), at which the sample size is deemed large enough for the detection of significant differences between groups. The data demonstrated a significant trend in NRF2 genotype-dependent concentration of pNRF2 in which NRF2 CC genotypes exhibited increased responses to the pharmacological NRF2 inducer DMF. 

When studying possible links between the parameters, we found a statistically significant positive correlation in the levels of Nrf2 protein and H2AX histone protein, a key component of DNA damage response, in the carriers of T+ rs35652124 genotypes (Table 4).

## 4. Discussion

### 4.1. Allelic Frequencies and Possible Clinical Significance

Literature search did not reveal reporting on Nrf2 SNP frequencies in infantile autism/ASD (PubMed and SFARI [45]). So, to our knowledge, our research was the first attempt to determine the possible association between ASD and functional NRF2 polymorphism. It is therefore expedient to juxtapose our findings with the reports from NRF2 polymorphism studies in diseases, which also affect the central nervous system, and the pathogenesis of which is also underpinned by impaired oxidative-antioxidative balance. First of all, they include neurodegenerative diseases (Parkinson’s disease, Alzheimer’s disease, amyotrophic lateral sclerosis), i.e., illnesses accompanied by massive cell death in conditions of chronic oxidative stress, also proven for autism/ASD (see Table 1). The set of principal data is shown below with respect to several NRF2 polymorphisms.

Published reports of the role and selective value of rs35652124 alleles are contradictory. Allele T of the promotor SNP rs35652124 in Far Eastern Asian populations (Taiwan, Japan) seems to be a risk allele: genotype TT was associated with hypertension and cardiovascular diseases (CVD). Possible associations of single nucleotide polymorphism rs35652124 (-653A/G) with biomedical measurements and lethality in patients, who had for a long time (9.10 ± 8.28 years) regularly undergone hemodialysis (HD) procedure, were analyzed in diabetic patients (119 male and 97 female patients). As a result, carriers of promotor T-allele rs35652124 were found to have increased CVD risk (OR 2.834; *p* = 0.006). Carriers of genotype AA (TT) in SNP rs35652124 had higher lethality from CVD compared to carriers of genotypes GG + GA (CC + CT) [29]. In Asians, allele C seems to provide for sufficient NRF2 content, while T does not, affecting among other targets reproductive health: SNP rs35652124 was found associated with oligoasthenozoospermia in Chinese patients, namely, subjects with genotype TT had a higher risk of this diagnosis [15]. The results of a cycle of studies performed by Japanese scientists suggest that NFE2L2 allelic variants are associated with tumorigenesis in the gastrointestinal tract [46] and risk of inflamed bowel syndrome caused by ulcerative colitis. Promotor haplotype rs35652124 C/rs6706649 C was reliably associated with an elevated frequency of p14 methylation, which is a hallmark of *Helicobacter pylori* infection (OR 2.90; 95% CI 1.14–7.36) [47]. A carrier state of alleles rs35652124 T/rs6706649 C was associated with higher scores of inflammation (*p* < 0.041) and a tendency for more severe atrophy of gastric mucosa [46]. In the Japanese population, haplotype rs35652124 C/ rs6706649 C appeared to be protective in regard to gastrointestinal diseases (OR 0.45; CI 0.22–0.93), while Japanese patients, who were heterozygous in each locus (rs35652124 C/T/rs6706649 C/T), were in a greater degree susceptible to ulcerative colitis (OR 2.57; CI 1.01–6.60) [18].

In Caucasians, however, TT might be a protective genotype in regard to Parkinson’s disease. In their research, von Otter with colleagues [19] studied promotor haplotypes (rs35652124 T/rs6706649 C/rs6721961 G) of the NFE2L2 gene in Swedish and Polish cohorts of patients with Parkinson’s disease. The promotor haplotype was found protective with respect to Parkinson’s disease (OR 0.6; CI 0.4–0.9). A meta-analysis of an expanded cohort (*n* = 1038), which included Italian, Maltese, and German patients, showed that the promotor allele rs35652124 C (G) increases an individual risk of Parkinson’s disease with earlier onset (the age of onset is decreased by −1.2 year; CI −2.12 to −0.02 year) [20]. The hypothetic difference in adaptive value of the alleles between Asian and European populations is indirectly corroborated by the difference in the frequencies of allele C (G): in Europe, it is a minor allele with a frequency not exceeding 0.25–0.35, supposedly, because of its negative selective value, whereas in Taiwan and Japan, where it is not associated with parkinsonism pathogenesis [48], its frequency reaches 0.4–0.6.

A recent study by Ran and colleagues [28] on the European population demonstrated that allele C (rs35652124) was protective with regard to the early onset of Parkinson’s disease (i.e., the presence of allele C delays the onset of clinical signs, hence, prevents its progression). Interestingly, notwithstanding the fact that this mutation affects the promotor region, no association of the patient’s genotype with the expression level of NRF2-specific mRNA was found, in the same manner as in our studies on children with ASD.

Perhaps, the peculiarities of a set (compound) of mutations that are included in a haplotype and have an opposite action towards the regulation of NRF2 production prevents in some cases correctly determining the link between expression level and a mutant site in the promotor region. In other words, another NRF2 mutation may alter the effect of the promotor variant allele C (NRF2 rs35652124).

Thus, the genetic heterogeneity of each of the disease and the resultant clinical heterogeneity of patterns particularly, age-dependent) are natural obstacles that prevent clarification of pathogenic predictive markers of progression of the ASD and other mental disorders.

### 4.2. Molecular Markers

When DNA damage appears due to genotoxic stress, phosphatidylinositol-3 family (PI3K-kinase family) kinases, such as ATM, ATR, and DNA-PK, phosphorylate H2AX to form so-called γ-focuses in DNA lesions to facilitate their repair. We found a significant correlation of protein Nrf2 and histone protein H2AX in the carriers of rs35652124 T+, but not CC genotypes. A study by Gruosso and coauthors [49] revealed a novel mechanism of the relation between the content of H2AX, free radicals (ROS), and Nrf2 in the cells of breast cancer (BC). A model was proposed that elevated ROS exhausted the intracellular pool of the adaptive key repair protein H2AX in chronic oxidative stress typical for BC cells. This process resulted in an increased susceptibility of BC cells to the action of anticancer agents and was mediated by the interaction of protein H2AX with E3 ubiquitine ligase RNF168, which led to proteasomal degradation of H2AX, thereby causing a stable reduction in H2AX in conditions of chronic oxidative stress. In view of the fact that high Nrf2 ensures active transcription of genes for antioxidant enzymes that quench free radicals, the above-mentioned mechanism of heightening susceptibility to genotoxic drugs is only effective in case of low Nrf2 expression. Therefore, in favorable conditions for continuing ROS generation (chronic oxidative stress), one can expect that cells with high NRF2 production would retain high H2AX content, whereas cells with genotypes, which does not provide a sufficient level of NRF2, would demonstrate reduced levels of H2AX.

There is a series of reports that an elevated generation of free radicals occurs in children with ASD [4]. The carriers of presumably protective genotype rs35652124 CC (in some reports it is marked as alternative chain, GG) produce enough NRF2 to secure ROS neutralization and avoid a drop of H2AX, whereas in the carriers of genotype rs35652124, T+ H2AX drops with an overabundance of ROS. The ROS dynamics were predicted to be cyclic [50]. Thus, such as in tumor cells [49], similar NRF-H2AX adjoint processes can occur in the immune system cells of children with ASD underpinning the positive correlation of contents of the two proteins we found in the study. At that, carriers of presumably protective genotype rs35652124 CC produce enough NRF2 to secure ROS quenching and avoid lowering H2AX, while in carriers of genotype rs35652124 T+, H2AX is high during low or moderate free radical levels, but drops for the time of ROS surplus periods.

Evidence for reduced Nrf2 expression, weakened aerobic respiration, and elevated generation of free radicals in ASD were also obtained on a small sample of children with ASD (*n* = 10) compared to healthy controls (*n* = 10) [51]. In children with ASD, Nrf2 expression was 45% of the reference level, however, genotypes of children with ASD were not studied in that research.

We found that induction of the phosphorylated (that is, active) form of pNRF2 protein was more impressive in carriers of genotype rs35652124 CC as compared to T+, though the significance of the difference was rather marginal (*p* = 0.049). Also, rs35652124 CC genotype demonstrated, compared to the other genotypes, a lower oxidation rate of circulating cell-free DNA (cfDNA) as determined by the percentage of 8-oxo-deoxyguanosine, which corroborates our data of a higher content of the phosphorylated form of NRF2 in the cells with this genotype. Moreover, in the blood plasma of carriers of genotype rs35652124 T+ we observed lower values of cfDNA/nuclease activity ratio. Low values of this ratio are inherent in chronic oxidative stress and exaggerated cell death [43]. In the aggregate, our findings suggest that genotype rs35652124 CC can be protective with respect to oxidative stress characteristic of the pathogenesis of autism.

Our findings demonstrate the potential for clinical significance of NFE2L2 gene polymorphism, which warrants further studies on linking clinically heterogeneous ASD with functional NFE2L2 gene polymorphism.

### 4.3. Nrf2 Induction in the Carriers of Different NFE2L2 Genotypes

Another important result of our study is substantiation of the pioneer perspective of personalized approaches to the treatment of patients with neurodegenerative (multiple sclerosis, parkinsonism, Alzheimer’s disease) and neurodevelopmental (autism/ASD) diseases, when the pathogenetically essential fact is insufficiency of antioxidation mechanisms, which could be compensated (at least, in a fraction of patients) by drug inducers of NRF2. Our findings open novel possibilities for the dependence of a significant stimulating effect of DMF on NRF2 functional polymorphism.

DMF is currently applied as a drug for the treatment of patients with multiple sclerosis. Few studies conducted in vivo [23,52,53] or in vitro [54] are focused on the prospect of treating children with ASD using another NRF2 inducer, sulforaphane. The studies report a temporary improvement of clinical parameters in a fraction of children. That is why our findings can suggest a possibility that the therapeutic effect of NRF2 inducers or boosters (dimethyl fumarate, sulforaphane, and others), if exists, can to the largest degree emerge in patients with genotype CC rs35652124, rather than CT or TT.

Our results suggest that following NRF2 induction with the drug, cells with NRF2 CC rs35652124 genotype can be boosted to a greater extent towards the active form of NRF2 protein. Our study has limitations such as one-time interval after the stimulation and limited cohort of patients, and the absence of age-matched controls to test the similar genotype association in non-ASD children. Certainly, much larger cohorts in future studies will provide more power to establish the most significant links of NRF2 genotypic differences in clinical responses to NRF2 inducers and reveal if NRF2 genetic polymorphism relates to the clinical heterogeneity of ASD course. 

Further studies are warranted to elucidate if the genotype-depending effect of NRF2 inducers is limited to the immune system cells of ASD patients or if the same dependence pertains to the cells of patients with diverse neurodegenerative diseases.

We would like to note finally that the genetic heterogeneity of ASD with more than 1000 genes involved [55] and the resultant clinical heterogeneity of signs (in particular, age-dependent) are natural hindrances for finding pathogenically significant predictive markers of ASD progression.

## Figures and Tables

**Table 1:** Single nucleotide polymorphism of Nrf2 was examined in the study and literature reports of its clinical value.

Code	Chromosome 2 Map Position (GRCh38.106) *	Location	Clinical Significance (on the Base of Published Literature)	Population of Origin
rs35652124	177265345 T > C	Promotor -214	Ulcerative colitis, gastritis, gastric ulcer	Japan [18,33]
			Cardiovascular mortality in hemodialysis patients	Japan [29]
			Arterial pressure, cardiovascular disease risk, and chronic obstructive pulmonary disease	Dutch [16]
			Parkinson’s disease	Sweden and Poland—meta-analysis [19,20], European countries [28]
			Oligoasthenozoospermia	China [15,34]

* Major allele (wild type) > minor allele.

**Table 2:** Allelic frequency distributions in six SNP sites in ASD cases and normal controls.

Genotype	rs35652124		
	T/T	C/T	C/C
Controls	32	52	16
Cases	60	90	29
P_compar._ (χ^2^)	*p* = 0.96		
Hardy-Weinberg, controls (χ^2^)	*p* = 0.501		
Hardy-Weinberg, cases (χ^2^)	*p* = 0.624		

**Table 3:** Comparison of the content of oxidation/genotoxic stress and adaptive response molecular biomarkers depending on NRF2 genotype and modulation with NRF2 inducer DMF.

	cfDNA/Nuclease Activity	NRF2	ph-NRF2	Nrf2 DMF/Baseline	ph-Nrf2 DMF/Baseline	8-oxo-G	mRNA Nrf2 DMF/Baseline	mRNA NQO1 DMF/Baseline	H2AX
CC	124.78 (92.95; 186.33)	2.055 (1.37; 2.79)	0.18 (0.14; 0.25)	0.98 (0.95; 1.49)	1.923 (1.653; 2.26)	0.66 (0.57; 0.76)	1.034 (0.63; 1.27)	0.924 (0.88; 1.07)	0.652 (0.51; 1.13)
CT	61.687 (24.64; 91.11)	1.832 (1.05; 2.74)	0.149 (0.08; 0.39)	1.389 (1.14; 1.54)	0.853 (0.46; 1.47)	1.08 (0.88; 1.20)	0.789 (0.73; 1.13)	1.107 (0.75; 1.28)	0.499 (0.37; 0.64)
TT	44.928 (29.38; 66.69)	2.72 (1.52; 3.17)	0.269 (0.14; 0.46)	1.196 (0.95; 1.48)	1.274 (0.69; 1.93)	1.18 (0.99; 1.51)	1.211 (0.88; 2.02)	1.034 (0.91; 1.44)	0.515 (0.47; 0.65)
C+	65.667 (35.97; 117.26)	1.88 (1.16; 2.79)	0.167 (0.09; 0.36)	1.349 (0.97; 1.53)	1.558 (0.85; 1.92)	1.01 (0.78; 1.15)	0.95 (0.72; 1.16)	0.97 (0.87; 1.16)	0.52 (0.41; 0.71)
T+	52.14 (26.08; 85.90)	1.99 (1.42; 3.11)	0.21 (0.09; 0.44)	1.35 (0.97; 1.51)	1.196 (0.68; 1.56)	1.12 (0.91; 1.34)	1.052 (0.75; 1.33)	1.058 (0.88; 1.33)	0.505 (0.41; 0.65)
CC vs. T+	*p* = 0.022 *	*p* = 0.860	*p* = 0.894	*p* = 0.703	*p* = 0.049 *	*p* = 0.018 *	*p* = 0.664	*p* = 0.270	*p* = 0.048 *
TT vs. C+	*p* = 0.317	*p* = 0.128	*p* = 0.235	*p* = 0.364	*p* = 0.792	*p* = 0.107	*p* = 0.156	*p* = 0.395	*p* = 0.861

Median value and, in parentheses, first and third percentiles (25%; 75%) are presented. *—significant difference (*p* < 0.05). Bonferroni correction was not applied. After incubation of cells with DMF drug, the mean content of Nrf2 protein and pNRF2 increased by a factor of 1.33 (0.97; 1.51) and 1.44 ((0.73; 1.93) versus baseline (same cell suspensions but prior to adding the drug), respectively, while transcription level of NRF2 and its target gene, NQO1, remained unchanged (ratios levels against background were 1.03 (0.75; 1.27) and 0.99 (0.83; 1.27), respectively).

**Table 4:** Spearman correlation of genotoxic and adaptive responses cells from different NRF2 rs35652124 genotypes.

Indices	Parameters	rs35652124 Genotype	
		CC	CT + TT
NRF2 vs. H2AX	R	−0.075	0.479 *
	P	0.809	0.0058
	N	9	53
8-oxo-G vs. pH2AX	R	0.24	0.169
	P	0.399	0.662
	N	14	8

R (Spearman rank correlation) values are presented. Interestingly, no correlation was observed in the carriers of the alternative genotype CC (Table 4).

## Data Availability

All data were included as a part of report #16-04-01541 A to Russian Foundation for Basic Research that had funded the study.

## Abbreviations

ARE—antioxidant response element; ASD—autism spectrum disorders; DMF—dimethyl fumarate; pNRF2—phosphorylated NRF2; SNP—single nucleotide polymorphism.
